# Highly Pathogenic Avian Influenza (H5N1): Pathways of Exposure at the Animal‐Human Interface, a Systematic Review

**DOI:** 10.1371/journal.pone.0014582

**Published:** 2011-01-24

**Authors:** Maria D. Van Kerkhove, Elizabeth Mumford, Anthony W. Mounts, Joseph Bresee, Sowath Ly, Carolyn B. Bridges, Joachim Otte

**Affiliations:** 1 MRC Centre for Outbreak Analysis and Modelling, Imperial College London, London, United Kingdom; 2 World Health Organization, Global Influenza Programme, Geneva, Switzerland; 3 Influenza Division, National Center for Immunization and Respiratory Diseases (NCIRD), Centers for Disease Control and Prevention, Atlanta, Georgia, United States of America; 4 Institut Pasteur du Cambodge, Phnom Penh, Cambodia; 5 Animal Production and Health Division, Food and Agriculture Organization of the United Nations, Rome, Italy; Universidade Federal de Minas Gerais, Brazil

## Abstract

**Background:**

The threat posed by highly pathogenic avian influenza A H5N1 viruses to humans remains significant, given the continued occurrence of sporadic human cases (499 human cases in 15 countries) with a high case fatality rate (approximately 60%), the endemicity in poultry populations in several countries, and the potential for reassortment with the newly emerging 2009 H1N1 pandemic strain. Therefore, we review risk factors for H5N1 infection in humans.

**Methods and Findings:**

Several epidemiologic studies have evaluated the risk factors associated with increased risk of H5N1 infection among humans who were exposed to H5N1 viruses. Our review shows that most H5N1 cases are attributed to exposure to sick poultry. Most cases are sporadic, while occasional limited human-to-human transmission occurs. The most commonly identified factors associated with H5N1 virus infection included exposure through contact with infected blood or bodily fluids of infected poultry via food preparation practices; touching and caring for infected poultry; consuming uncooked poultry products; exposure to H5N1 via swimming or bathing in potentially virus laden ponds; and exposure to H5N1 at live bird markets.

**Conclusions:**

Research has demonstrated that despite frequent and widespread contact with poultry, transmission of the H5N1 virus from poultry to humans is rare. Available research has identified several risk factors that may be associated with infection including close direct contact with poultry and transmission via the environment. However, several important data gaps remain that limit our understanding of the epidemiology of H5N1 in humans. Although infection in humans with H5N1 remains rare, human cases continue to be reported and H5N1 is now considered endemic among poultry in parts of Asia and in Egypt, providing opportunities for additional human infections and for the acquisition of virus mutations that may lead to more efficient spread among humans and other mammalian species. Collaboration between human and animal health sectors for surveillance, case investigation, virus sharing, and risk assessment is essential to monitor for potential changes in circulating H5N1 viruses and in the epidemiology of H5N1 in order to provide the best possible chance for effective mitigation of the impact of H5N1 in both poultry and humans.

**Disclaimer:**

The opinions expressed in this article are those of the authors and do not necessarily reflect those of the institutions or organizations with which they are affiliated.

## Introduction

There have been several human pandemics caused by influenza A viruses over the last 150 years [1], [2], [3]. The first pandemic of the 20^th^ century, the “Spanish” influenza (H1N1) pandemic of 1918–1919, was particularly lethal in young, otherwise healthy adults, killing an estimated 40–50 million people worldwide [2], [4], [5], [6]. Genetic analyses of specimens collected from victims preserved in the arctic and archived tissues from World War I soldiers suggests that the 1918 H1N1 strain was an avian-origin virus that adapted to humans [7]. The “Asian” influenza pandemic (H2N2) in 1957 and the “Hong Kong” influenza pandemic (H3N2) in 1968 were less lethal and resulted from avian-human virus reassortment [4], [5]. The 2009 H1N1 pandemic influenza virus is a reassortant of human, swine and avian-origin influenza virus gene segments, with the HA gene sharing a common ancestry with the 1918 pandemic virus HA that has been circulating in swine populations globally [8]. Since its emergence in the spring of 2009, the pandemic H1N1 virus quickly became the predominant strain globally[9].

The isolation of a highly pathogenic avian influenza A virus, subtype H5N1 (referred to as H5N1 in this manuscript), from a 3-year-old boy in Hong Kong in 1997 was the first detection of this virus strain in humans and raised concerns worldwide of the potential for a pandemic of avian origin with a lethality in the range of the 1918 pandemic [10]. As with the 1918 virus, all of the genes found in the H5N1 viral strain in Hong Kong originated from avian viruses, [4], [10]. While H5N1 has not yet demonstrated the ability to transmit efficiently from person to person, the high case-fatality associated with infection, and because of the immense potential for influenza viruses to mutate and adapt to other hosts, H5N1 remains a continuing public health concern.

As of 8 June 2010, 15 countries have reported a total of 499 confirmed cases of human H5N1 infection to WHO [11]. By far, the largest numbers of human cases has been reported from Indonesia, Vietnam and Egypt, each having reported more than 100 cases (these three countries account for 79% of all human cases). No human cases have yet been reported in Western Europe or the Americas, although H5N1 has been detected in poultry in Europe. The number of reported cases and fatalities, case fatality rate (CFR), H5N1 virus clades identified that have infected humans, and the median age and gender (% male) of reported cases [12], [13] vary by country (Table 1). The crude CFR for all cases to date is high (CFR = 59.1%, interquartile range 32.5–77.8), but also varies substantially among the 15 countries.

**Table 1:** Characteristics of human cases of highly pathogenic avian influenza H5N1 virus infection reported to WHO from 1997 to 16 March 2010 by country.

Country	Total		Crude CFR (%)	Clade(s)^A^	Median age of cases (range)^B^	% Male n/total (%)^B^
	Cases	Deaths				
Azerbaijan	8	5	62.5	2.2	10 16.5 (5–20) ‡‡	9/16 (56) ‡‡
Turkey	12	4	33.3	2.2		
Bangladesh	1	0	0.0	2.2	16 mo (–)	1/1 (100)
China	38	25	65.8	2.2, 2.3.4, 7	30 (12–41)‡	3/8 (38)‡
Hong Kong, SAR (1997)	18	6	33.3	0, 1	6 (1.5–60)	6/15 (40)
Djibouti	1	0	0.0	2.2	2 (–)	0/1 (0)
Egypt	106	32	30.2	2.2	12.5 (1–75)α	12/38 (32)α
Indonesia	163	135	82.8	2.1.2, 2.1.3	18.5 (1.5–45)‡	33/54 (61)‡
Iraq	3	2	66.7	2.2	15 (3–39)	2/3 (66.7)
Lao People's Democratic Republic	2	2	100.0	2.3.4	28.5 (15–42)	0/2 (0)
Myanmar	1	0	0.0	2.3.4	7 (–)	0/1 (0)
Nigeria	1	1	100.0	2.2	22 (–)	0/1 (0)
Pakistan	3	1	33.3	NR	25 (22–27)	3/3 (100)
Cambodia	9	7	77.8	1	14–22 (2–58)†	19/41 (46)†
Thailand	25	17	68.0	1		
Vietnam	116	58	50.0	1, 2.3.4		
Total	489	289	59.1	–	–	–

A Clade(s) isolated from humans.

B Data from cases up to 1 Jan 2009.

Adapted from sources [13], [17], [63], [64], [65], [66], [67].

† Includes data from 2004–2005 cases only;

‡ Includes data from 2005–2006 cases only;

α Includes data from 2006–2007 cases only;

‡‡ Includes data from 2006 cases only;

NR =  Not released.

To date H5N1 remains an avian epidemic with sporadic spill-over into the human population and other species. The predominant modes of transmission from poultry to humans remain incompletely understood and limited exposure information from infected persons has restricted our ability to evaluate risk factors for human infection and implement more refined risk reduction measures. Field investigations of cases of H5N1 in humans—usually in locations of low or middle income countries—are generally difficult to conduct, especially in a timely manner, and may result in collection of incomplete exposure information. Conversely, in some countries, good exposure data has been collected during outbreak investigations, but may not be analyzed or published. Thus, information on potential exposures, when given, is typically limited to *recent contact with sick or dead poultry* [14] or the *preparation of sick birds for consumption* [15]. More detailed knowledge of the types of behaviors and interactions with poultry that result in virus transmission would facilitate more effective and targeted risk reduction measures at the human-animal interface.

Several epidemiologic studies have been published to evaluate risk factors, including contact with poultry and poultry products and non-poultry-related contact such as from H5N1-contaminated water, for H5N1 infection in humans. Most of these have adopted a case-control (or nested case-control) design where researchers have evaluated the risk of exposure to poultry from visiting live bird markets (LBM), food preparation, caring or feeding poultry or exposure risk via contact with a confirmed human case. In 2009, Rabinowitz et al. published a systematic review of published analytical studies and case reports through 2007 on exposure variables for human cases of H5N1 infection. Since this publication, a number of published large-scale seroprevalence studies in areas where H5N1 has occurred or is recurrent have been published. Here we evaluate what is known about pathways of exposure at the animal-human interface using all available publications, including seroprevalence studies and case-control studies not included in previous reviews, which could result in human infection with H5N1 virus.

## Methods

A systematic search for all available published literature evaluating prevalence of symptomatic or asymptomatic infection with H5N1 and/or risk factors for human infection with the H5N1 virus was performed in MEDLINE using the following keywords together and in various combinations: “H5N1, risk factor, poultry, seroprevalence, antibodies, human, animal-human interface”. All papers published between 1 January 1997 and 1 April 2010 are included in the review regardless of the language of publication. The original search yielded 444 articles. All titles were reviewed to identify epidemiologic studies that evaluated risk factors among human populations. The abstracts were reviewed for papers from which a decision could not be made from the title alone. Case reports, vaccine efficacy studies, laboratory studies and studies in animal populations were excluded from this review. This review updates a previous review by Rabinowitz et al [16], using studies published between 2008–2010.

Twenty-four published studies evaluating risk and/or risk factors for human infection conducted in 8 countries (Thailand, Vietnam, Indonesia, Cambodia, Nigeria, China, Azerbaijan, and Germany) and Hong Kong were included in the review. Four studies focused on the initial 1997 outbreaks in Hong Kong, while the remaining 20 studies were conducted in Asian, African and European countries in areas with confirmed outbreaks in human and/or domestic poultry populations from 2003–2009. Based on the population under study and principal objective, the 24 studies fall into two categories: case-control studies to evaluate risk factors for human infection among laboratory-confirmed H5N1 cases (n = 5; 2 related to the 1997 outbreak and 3 related to outbreaks occurring 2003 to 2009); or seroepidemiology studies (n = 19; 3 relating to the 1997 outbreak and 16 related to outbreaks occurring 2003 to 2009) to evaluate the predictors of having H5-specific antibody among health care workers (HCW; n = 4), poultry workers (PW; n = 8) or household/social contacts (n = 8) of laboratory-confirmed infected H5N1 cases (one study evaluated both occupational and domestic exposure to poultry and is therefore counted as both a study among PW and social contacts).

## Results

### Investigations into the 1997 H5N1 outbreak in Hong Kong (18 cases, 6 deaths)

The H5N1 virus was first known to cross the animal-human species barrier in 1997 when 18 hospitalized, symptomatic cases, six of whom died, were identified in Hong Kong [10]. A case-control study of 15 of these confirmed H5N1 cases and 41 controls matched on age, sex and neighborhood found that exposure to live poultry at LBM in the week before illness was associated with a 4-fold increased risk in infection (OR = 4.5 95%CI 1.2–21.7) (Table 2). No association was found with consumption of cooked or undercooked poultry at home or at a restaurant [17].

**Table 2:** Risk factors for H5N1 infection: Summary of published case-control studies.

Study, year	Study Population	Risk FactorsRR, OR, 95%CI
Mounts et al., 1999 [17]	Hong Kong15 cases 41 matched controls	Exposure to poultry at live/wet markets was associated with a 4-fold increased risk (OR = 4.5, 1.2–21.7)
Dinh et al., 2006 [23]	Viet Nam28 cases 106 matched controls	Univariate Analysis: preparing/cooking unhealthy poultry (OR = 31, 2.4–1150), having sick or dead poultry in the household (OR = 7.41, 2.7–59), presence of sick/dead poultry in the neighborhood (OR = 3.9, 1.0–55.7), no indoor water source in the household (OR = 5.0, 1.3–77.0)Multivariate Analysis: No water in the household (OR = 6.5, 1.2–34.8), sick or dead poultry in the household (OR = 4.9, 1.2–20.2), prepare and cook sick or dead poultry (OR = 9.0, 0.98–82.0)
Areechokchai et al., 2006 [22]	Thailandmatched case control study of 16 cases and 64 controls	Direct touching of unexpectedly dead poultry OR 29.0 (2.7–308.2)
Zhou et al., 2009 [39]	China10 urban and 18 rural cases; 134 matched controls	Infection included direct (OR = 506.6, 95%CI15.7–16319.6) or indirect (OR = 56.9, 95%CI 4.3–745.6) contact with sick or dead poultry, visiting a LBM (OR = 15.4, 95%CI 3.0–80.2)Urban cases were significantly more likely to have visited a LBM, compared with rural cases (p = 0.002)
WER, 2006 [33]	Azerbaijan, residents in settlements of confirmed cases	9/52 residents tested positive for H5N1 virus.No case-control was initiated, but contact with infected wild birds (defeathering) reported as likely cause of infection

The extent of anti-H5 seroprevalence was evaluated among household/social contacts [18], HCW caring for confirmed human H5N1 cases [19], and PW involved in the culling of all poultry in Hong Kong (Table S1; in supplemental information) [20]. Six of 51 (12%) household contacts and none of 26 social contacts tested positive for anti-H5 antibodies using microneutralization (MN) and Western Blot (WB) techniques[18]. Although not statistically significant, the authors of this study suggest that common-source exposure of the household contacts to poultry in their homes was a likely risk factor for infection. Among HCW, risk factor data were collected including exposure to the case patient (e.g. provided direct care to case, physical contact, face-to-face talking, worked within two meters of patients, recalled patient coughing/sneezing, suctioned respiratory secretions from or administered breathing treatments to patients, changed bed linens or bathed the patient), age, sex, occupation and exposure to poultry (shopped at live poultry market, had live or freshly cut poultry in their home in the weeks before interview). Because the initial diagnosis was delayed, infection control procedures were not immediately initiated for most cases. Among the exposed and unexposed HCW enrolled, 4% (8/217) and 0.7% (2/309), respectively, tested positive for H5 antibodies, suggesting a risk of patient to HCW transmission. Exposure to poultry did not differ among exposed and unexposed HCW. Risk factors for H5 antibody among exposed HCW included bathing the patient and changing bed linens, tasks that involve close and more prolonged exposure to the patient. Interestingly, no HCWs exposed to mildly ill children had anti-H5 antibodies, only HCW exposed to critically ill patients with pneumonia, both of whom died, had H5 antibody.

Among 1,525 PW and among 293 government workers (GW) who were involved in the culling of poultry during this outbreak in Hong Kong, 10% of PW were estimated to be seropositive to H5, while 9 (3.1%) GW tested positive [20]. A nested case-control study of PW found an elevated risk for those that worked in retail compared to those who worked in wholesale, hatchery, farm, or other poultry industries (OR = 2.7 95% CI 1.5–4.9); worked on a farm with >10% mortality among poultry within the previous two months (OR = 2.2 95% CI 1.3–3.7); butchered poultry (OR = 3.1 95% CI 1.6–5.9); fed poultry (OR = 2.4 95% CI 1.4–4.1); and prepared poultry for restaurants (OR = 1.7 95% CI 1.1–2.7). The risk of having anti-H5 antibody appeared to increase with the amount and intensity of contact with poultry, with stratified analysis suggesting that butchering poultry and exposure to poultry flocks with >10% mortality were exposures most highly associated with having anti-H5 antibody. Feeding poultry was not associated with an increased risk in stratified analyses.

### Sero-epidemiological investigations since 2003 (499 cases, 295 deaths)

Since 2003, sero-epidemiologic investigations into risk factors for human infection have been conducted primarily in Asian countries and to a lesser extent in African, European and the Middle Eastern countries (Table S1) but human seroprevalence studies have not been conducted in all locations with relatively high numbers of human cases (e.g., Egypt, Vietnam and Indonesia). Rather, several small scale studies evaluating the prevalence of anti-H5 antibodies have been conducted in Vietnam, Thailand, Cambodia, China, Indonesia, Germany, and Nigeria in areas (within 1–3 km) surrounding locations of reported human and/or poultry outbreaks [21], [22], [23], [24], [25], [26], [27], [28], [29], [30], [31], [32], [33], [34], [35]. These sero-studies can be categorized by the study populations evaluated in each study: non-occupational settings (subjects living in close proximity to a confirmed H5N1 case) and occupationally exposed individuals (PW or HCW) (Table S1).

#### Non-occupational settings

Non-occupational exposure largely consists of caring for household poultry, preparing or cooking poultry, visiting a LBM or living in close proximity to poultry. Three studies from Thailand, Cambodia and Indonesia of the seven studies evaluating seroprevalence in rural areas found no evidence of anti-H5 antibodies in their study populations despite frequent contact in households with poultry with probable H5N1 infection [24], [29], [30]. However, evidence of exposure to poultry resulting in asymptomatic human infection was found in 1 study in China, and 2 studies in Cambodia. In the studies from Guangdong China and Cambodia, approximately 1–3% (14/1214[25], 7/674 [36], 18/700 [37]) of the individuals living within a 3 km or 1 km radius, respectively, of H5N1 outbreaks in domestic poultry had antibodies against H5 indicating prior infection with H5N1. In Cambodia, risk factors associated with seropositivity included swimming or bathing in ponds (OR = 11.3, 95% CI 1.25–102.18 [36]; OR = 2.52, 95%CI 0.98–6.51[37]) and gathering poultry and placing them in cages or designated areas (OR = 5.8, 95% CI 0.98–34.12[36]).

Two case-control studies were conducted in Vietnam (28 cases; 106 age-, sex-, and neighborhood- matched controls [23]) and Thailand (16 cases, 64 age- and neighborhood-matched controls [22]; Table 2). Using multivariate analysis, the Vietnam study found that risk factors for human infection included preparing or cooking unhealthy poultry (OR = 31, 95%CI 3.4–1150), having sick or dead poultry in the household (OR = 7.41, 95%CI 2.7–59.0), presence of sick/dead poultry in the neighborhood (OR = 3.9, 95%CI 1.0–55.7), and no indoor water source in the household (OR = 5.0, 95%CI 1.3–77.0) [23]. In Thailand, cases were more likely to have: touched a dead bird that died unexpectedly (OR = 29, 95%CI 2.7–308.2); dressed poultry (no definition provided, OR = 17, 95%CI 1.6–177.0); had poultry that died unexpectedly around their home (OR = 5.6, 95%CI 1.5–20.7); plucked feathers from poultry (OR = 14, 95%CI 1.3–152.5); stored products of sick or dead poultry in their home (OR = 9.3, 95%CI 2.1–41.3); or directly touched sick poultry (OR = 5.6, 95%CI 1.5–20.7). Risk factors for infection also included living in close proximity to sick (OR = 3.8, 95%CI 1.2–11.7) or dead (OR = 13, 95%CI 1.5–96.3) poultry [22]. Following an outbreak of H5N1 in wild birds in Azerbaijan in 2006, the clinical specimens (throat, nasal and rectal swabs, plus sera) of 9/52 residents (all symptomatic) tested positive for the presence of H5N1 virus using RT-PCR and virus isolation. These 9 cases, all of whom were from related or neighboring families, were thought to most likely have become infected while defeathering dead wild swans [38].

In China, a case-control analysis of 10 urban and 18 rural laboratory confirmed human H5N1 cases compared to 134 controls found that risk factors for infection included touching sick or dead poultry (OR = 506.6, 95%CI15.7–16319.6) or living in close proximity to sick or dead poultry (OR = 56.9, 95%CI 4.3–745.6), and visiting a LBM (OR = 15.4, 95%CI 3.0–80.2) [39] (Table 2). Urban cases were significantly more likely to have visited a LBM, compared with rural cases (p = 0.002).

#### Occupational exposure

Risk factors for infection among PW at LBMs or workers involved in culling operations have been evaluated in Nigeria, China (Guangdong), Indonesia (Bali), Vietnam and Germany. Despite presumably frequent and extensive contact with infected poultry, no evidence of H5N1 infection was found among 295 market vendors in Nigeria [26], 87 market vendors in Bali [30], 68 market vendors in Guandong, China [40], or 97 GW involved in culling operations in Germany [31]. Three studies from Guangdong, China (1 seropositive/110 tested using HI with turkey red blood cells[35]; 2/231 using HI>1∶80[25]; 2/2191 using HI [no cutoff mentioned]) and one study from Vietnam (3 seropositive/500 tested using HI>1∶80, 0/500 using MN) found limited evidence of previous H5N1 infection; however, no specific risk factors for infection were reported (Table S1) [25], [32], [35], [41].

Since 2003, one study from Thailand, and two studies from Vietnam evaluated the frequency of asymptomatic or subclinical infection and evaluated human-to-human transmission risk factors for H5N1 virus among HCW [21], [27], [28]. In contrast to the results found in the serosurvey of HCW conducted in Hong Kong in 1997[19], no serologic evidence was found of infection with H5N1 among HCW with direct contact with human H5N1 patients. The use of personal protective equipment (PPE) in Vietnam was well documented [27], [28]. In Thailand, however, the use of PPE (surgical mask, gown and gloves) was not initiated until 48 hours after the patient was admitted to the hospital [21].

Person to Person transmissionClusters of epidemiologically linked H5N1 cases have occurred among relatives in several countries, including Indonesia, China, Turkey, Azerbaijan, Vietnam and Thailand, suggesting that human-to-human transmission between family members in close contact may have occurred [38], [42], [43], [44], [45], [46], [47], [48]. An early investigation in Vietnam, suggested that between January 2004 and July 2005, 15 suspected family clusters occurred among the first 109 cases, of which nine clusters had ≥2 laboratory confirmed H5N1 cases [42].

A family cluster in mainland China consisted of a father and son, the former likely infected through close, unprotected contact via care of his son at a hospital during his illness [46]. Similarly in Thailand, two relatives of an infected patient likely became infected through unprotected hospital care [44]. In Turkey, several members of the same family became infected with H5N1, however transmission was likely common-source poultry-to-human rather than human-to-human because they all shared the same living space with poultry [43].

In Indonesia, there have been reports of 21 clusters of H5N1 among blood relatives with each cluster involving 2–7 blood relatives [45], [47], [48]. Limited human-to-human transmission may have occurred in two of the first three clusters in 2005. However, common-source exposure to the virus via a contaminated environment, through contact with contaminated poultry manure or with infected poultry could not be ruled out [45]. In a further detailed analysis of all human H5N1 cases in Indonesia, the authors examined exposures to poultry and could not rule out a common source of infection in the clusters as family members usually have similar opportunities for exposure to the virus.

### Environmental exposures leading to transmission of H5N1 virus to humans

Non-poultry exposures-related H5N1 exposures, defined here as any contact not involving touching poultry or poultry products, e.g. exposure to H5N1 contaminated environments may also lead to H5N1 infection [36], [49], [50], [51], [52]. Exposure to H5N1 virus in contaminated feces in garden fertilizer has been reported as a source of human infection [53]. Because birds are known to shed high concentrations of virus into water sources, transmission from poultry to humans through contaminated water is also possible [52]. The epidemiologic investigation of two H5N1 cases in a single family in Vietnam suggested that exposure to possibly contaminated canal water via swimming or washing may have resulted in infection. However, the role of water in transmission could not be confirmed [49]. More recently, results from environmental sampling within a Cambodian village with confirmed H5N1 in domestic poultry flocks and one human case as well as results from a human seroprevalence study from the same village identified contaminated water as a potential risk factor for H5N1 infection [36], [51].

## Discussion

Several epidemiologic studies have been published to evaluate risk factors, including contact with poultry and poultry products and non-poultry-related contact such as from H5N1-contaminated water, for H5N1 infection in humans. Our review shows that most H5N1 cases are attributed to exposure to sick poultry, while a few were likely due to human-to-human transmission.

**Figure 1:**
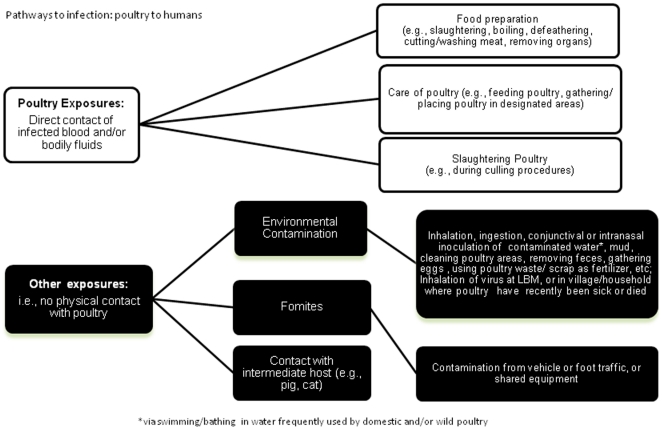
Known and suggested pathways of H5N1 exposure to infection from poultry to humans. *via swimming/bathing in water frequently used by domestic and/or wild poultry.

An illustration of possible pathways of poultry-to-human transmission of H5N1 virus is provided in Figure 1. Potential modes of influenza transmission vary depending on the nature of the contact, and have been suggested to include inhalation; ingestion; conjunctival, oral contact or intranasal inoculation; or aerosol routes [16]. Evidence from the published literature has illustrated that exposure to the H5N1 virus has occurred through contact with infected poultry blood or bodily fluids via food preparation practices [54] (e.g., slaughtering, boiling, defeathering, cutting meat, cleaning meat, removing and/or cleaning internal organs of poultry); consuming uncooked poultry products (e.g., raw duck blood) [21], [49], [55] or through the care of poultry (either commercially or domestically) [36]. The extent and frequency of risk behaviors and the relative risk of different behaviors is currently unknown across all countries where H5N1 is recurrent or endemic and there may be reluctance to disclose information on possible individual exposures due to legal, social or economic implications, or other reasons. For example, in Azerbaijan the nine human cases were likely exposed during the illegal de-feathering of dead wild swans [38].

There are also a significant number of human H5N1 cases reported to WHO without known or reported poultry exposure [56]. Little is understood about non-direct contact exposures to H5N1-infected poultry that may increase the risk of human infection, though recent studies have suggested an association between exposure to a contaminated environment (e.g., water; cleaning poultry cages or their designated areas; using poultry feces for fertilizer) and infection either through ingestion, conjunctival or intranasal inoculation of contaminated water, soil [49], [51], [53] or via fomites e.g. equipment or vehicles [50]. It is also possible that infection via inhalation of H5N1 aerosolized at LBMs in China may have occurred [17], [39]. Other pathways may exist, but are currently unknown.

The collective results of publically available studies have shown that transmission of H5N1 virus from poultry to humans is infrequent, given that often only a single case may be detected in an area with widespread illness and death among household poultry, for example. Furthermore, the nature of the contact between some H5N1 patients and poultry was extensive, i.e., via preparing infected poultry, while some cases have reported less intense exposure to virus such as during swimming or bathing in potentially virus laden ponds or visiting LBMs without direct contract with poultry, and some have had no known exposure to poultry prior to infection [53], [57]. A better understanding of the risk of transmission via direct or indirect contact, through ingestion or inhalation or other exposure routes is needed to refine strategies to reduce risk of H5N1 infections in people.

It is highly likely that types of human-poultry contact differ between and even within countries. For example, there is substantial variation in the frequency of different poultry contact practices (e.g., slaughtering, caring for poultry) by age and gender amongst populations in rural Cambodia living in close proximity to poultry [58]. Research has demonstrated that, based on reported poultry contact patterns, males in rural Cambodia have a higher exposure risk potential to H5N1 than females across all age groups and exposure risk is highest among males between the ages of 26–40, followed by 16–25 years old. Males in these age groups reported practices of contact with poultry (e.g., slaughter poultry, remove internal organs, blow in the beak of fighting cocks, clean the trachea of fighting cocks, lick wounds of fighting cocks) that give rise to the highest H5N1 transmission risk potential [58]. Such differences demonstrate that the potential risk for transmission of H5N1 from poultry to humans is not uniform across age and gender and therefore may not be uniform within or across countries. The demographic differences in human cases of H5N1 infection to date among countries (Table 1) are likely because such contact patterns with poultry—in addition to animal husbandry practices, biosecurity systems for the production of food animals and systems for detection of clinical disease—also differ among countries. However, data could also suggest that the variation in H5N1 incidence by age may not be due to exposure alone and that there may be differences by age in susceptibility to infection, pre-existing immunity against human influenza viruses that may confer some cross-reactive immunity, clinical presentation of disease, and/or presentation to health care facilities. In some countries, inclusion of contact with sick poultry in the definition of a suspect case could lower the case detection rate as well as falsely increase the proportion of cases with exposure to sick poultry as a risk factor. Additionally, ascertainment and recall biases could have been introduced in exposure assessment due to media coverage and/or lengthy delays between reported human and/or poultry H5N1 cases and follow-up epidemiologic investigations.

Our results also demonstrate a difference in seropositivity rates among serosurveys conducted following the 1997 H5N1 outbreaks in Hong Kong when compared to serosurveys conducted following outbreaks from 2003 to 2010. The higher rates of seropositivity in the studies following the 1997 outbreak may reflect the genetic differences in the viruses circulating now compared to the 1997 virus, which may have been more adaptable to human infection [59]. Sustained vigilance is required to monitor the ever changing nature of these viruses.

Several important data gaps currently limit our understanding of the transmission of H5N1 from poultry or H5N1 contaminated environments to humans. First, there is likely some unknown level of underreporting of human cases and poultry outbreaks such that the range and types of exposures may differ from reported cases. There may also be data and analyses conducted on H5N1 cases that have not been made publically available. Second, the serologic studies were conducted by different laboratories using a variety of assays and cutoffs for seropositivity, making direct comparisons of results across studies difficult. Seroprevalence studies have identified few asymptomatic individuals with anti-H5N1 antibodies, indicating previous infection with H5N1. However, the duration of immunity after H5N1 infection is not known and the timing of sampling in these studies may have resulted in an underestimation of those having experienced prior infection. In addition, it is possible that some infected individuals may not seroconvert and that some antibody positive individuals have non-specific antibody against H5 and do not represent true prior infections.

Third, the influence of genetic and/or immunological factors on susceptibility to infection and disease is poorly understood. Although there have been several suspected clusters of H5N1 infection largely among blood relatives [42], [43], [44], [45], [46], the clusters are difficult to interpret because not all potentially exposed family members may have been tested for H5N1 and in most clusters, a common exposure could not be ruled out. While there may have been limited human-to-human transmission among close contacts in some clusters, genetic variation between families could result in the occurrence of clusters because of a predisposition to infection [47], [60].

Finally, improved knowledge is needed on potential routes of transmission of H5N1 virus from poultry or H5N1-contaminated environments to humans and on the prevalence of risky practices in human populations. Studies to date have evaluated exposures through which people might become infected with H5N1, but we currently lack sufficient data from the confirmed H5N1 cases around the world and published epidemiologic studies to fully evaluate other potential risk factors for infection such as the role of water and other environmental factors, or unknown risk factors that have yet to be investigated. Transmission routes could also include oral ingestion, conjunctival or intranasal inoculation from contaminated water while drinking, swimming or bathing or inhalation of the virus in feces while caring for poultry [36]. Furthermore, more asymptomatic cases may occur because of low concentrations of viruses in the environment than have been detected in studies done to date. More studies of environmental contamination, including viral contamination in LBMs [61], would further contribute to this understanding.

In order to fully evaluate the risk of poultry-to-human transmission, a detailed exposure history needs to be collected from all suspected cases and their contacts. In addition, data variables related to exposures to poultry by species and potentially infected environments ideally should also be standardized across epidemiologic studies to facilitate pooled or meta-analyses. Data collection forms have been developed [62]; however, these forms must include not only information on contact with poultry by species, but include questions regarding the timing and intensity of such contact. These forms should also not only evaluate general exposure (e.g., handling sick or dead poultry, handling feces or fertilizer from sick or dead poultry, slaughtering poultry), but should include potential exposure via the environment (e.g., contaminated water). In order to build a database from which more robust analysis can be conducted, detailed exposure information should be systematically collected from all confirmed and suspect cases.

Although infection in humans with H5N1 virus remains rare, human cases continue to be reported. As well, H5N1 is now considered endemic among poultry in parts of Asia, providing opportunities for further dissemination of this virus and opportunities to mutate and adapt to humans and other mammalian species. Collaboration between human and animal health sectors for surveillance, case investigation, virus sharing and risk assessment is essential to understand and reduce the risk of virus transmission at the interface between domestic poultry and humans and to quickly recognize changes that may occur in the virus or in the epidemiology of its spread to humans that signal adaptation to humans. Current exposure data remain too general to explain the current pattern or to predict future cases of H5N1 infection in human populations; however the results of the available studies, including those reporting cases having no contact with poultry, suggest that exposure through the environment may account for many human cases [36], [39]. Rapid, systematic and standardized collection of detailed information on poultry contact and human case contacts for all suspected and confirmed human cases of H5N1, as well as more systematic epidemiological and seroepidemiologic studies with appropriate controls, would improve our understanding of risks of H5N1 and help inform development and implementation of appropriate public health risk reduction measures.

## Supporting Information

Table S1 Results of seroprevalence studies to determine the frequency of asymptomatic or subclinical infection and evaluate risk factors for H5N1 virus infection.
(0.50 MB PDF)
