# Correction: Highly Pathogenic Avian Influenza (H5N1): Pathways of Exposure at the Animal‐Human Interface, a Systematic Review

**DOI:** 10.1371/annotation/3531c496-624f-40fe-92bb-5fb3d7d1d894

**Published:** 2012-01-03

**Authors:** Maria D. Van Kerkhove, Elizabeth Mumford, Anthony W. Mounts, Joseph Bresee, Sowath Ly, Carolyn B. Bridges, Joachim Otte

The abstract of the article cited consumption of uncooked poultry products as one of the most commonly identified factors associated with H5N1 virus infection. While this factor has been previously mentioned in the literature, it was not identified as such as part of the results of the systematic review reported in the article.

Given that the consumption of uncooked poultry products was not identified as a risk factor associated with H5N1 virus infection as part of the Results reported in the article, the reference to this in the abstract may lead to confusion. The authors are therefore publishing this Correction in order to remove the statement related to consumption of uncooked poultry from the abstract.

