# Supplementary material for: Highly Pathogenic Avian Influenza (H5N1): Pathways of Exposure at the Animal‐Human Interface, a Systematic Review
Source: PLoS One. 2011 Jan 24;6(1):e14582. doi: 10.1371/journal.pone.0014582 (PMC3025925; doi:10.1371/journal.pone.0014582)
Supplement: Table S1 — Results of seroprevalence studies to determine the frequency of asymptomatic or subclinical infection and evaluate risk factors for H5N1 virus infection. (0.50 MB PDF) [file pone.0014582.s001.pdf]

Supplemental Information:

Highly Pathogenic Avian Influenza (H5N1): Pathways of Exposure at the Animal-Human Interface, a systematic review

Maria D Van Kerkhove[1*], Elizabeth Mumford[2], Anthony W Mounts[2], Joseph Bresee[3], Sowath Ly[4], Carolyn B. Bridges[3], Joachim Otte[5]

[1] MRC Centre for Outbreak Analysis and Modelling, Imperial College London, London, United Kingdom
[2] World Health Organization, Global Influenza Programme, Geneva, Switzerland
[3] Influenza Division, NCIRD, Centers for Disease Control and Prevention, Atlanta, GA, USA
[4]Institut Pasteur du Cambodge, Phnom Penh, Cambodia
[5] Animal Production and Health Division, Food and Agriculture Organization of the United Nations, Rome Italy

*Corresponding author:
Maria D. Van Kerkhove, PhD
MRC Centre for Outbreak Analysis and Modelling
Department of Infectious Disease Epidemiology
Imperial College Faculty of Medicine
St Mary's Campus, Norfolk Place
London W2 1PG, United Kingdom
Tel: +44 (0)75 902 50513
Email: m.vankerkhove@imperial.ac.uk

Table S1 Results of seroprevalence studies to determine the frequency of asymptomatic or subclinical infection and evaluate risk factors for H5N1 virus infection

Study, year	Study Population & Year of Outbreak	Transmission	Seroprevalence Results (% seropositive)	Risk Factors RR, OR, 95%CI	Comments
Occupationally Exposed Persons: Poultry Workers					
Bridges et al., 2002 (1)	Poultry workers, Hong Kong 1997	Poultry-to-humans	9/293 (3%) GW were seropositive<br>10% PW were estimated to be seropositive using MN >80<br>Nested case-control study conducted among 81 seropositive cases and 1231 controls	Work in retail vs. wholesale/ hatchery/farm/other poultry industry 2.7 (1.5-4.9)<br>>10% mortality among poultry 2.2 (1.3-3.7)<br>Jobs: -Butchering poultry 3.1 (1.6-5.9)<br>· Handling money 1.6 (1.0-2.5)<br>· Preparing poultry for restaurants 1.7 (1.1-2.7)	Limited poultry-to-human transmission among PW and GW involved in poultry culling operations
Wang et al., 2006 (2)	Poultry workers, Guangdong China, 2006	Poultry-to-humans	1/110 PW were seropositive using HI with turkey erythrocytes >320	Specific risk factors not identified, but subject slaughtered poultry for 5 years	Specific risk factors not identified
Oritz et al., 2007 (3)	Poultry workers, Kano Nigeria<br>2006	Poultry-to-humans	0/295 PW with median 14 days exposure to H5N1<br>0/25 laboratory workers with exposure to H5N1<br>Seropositivity by MN titers if ≥1:80	None	No evidence of H5N1 infection with subjects with repeated exposure to infected poultry
Lu et al., 2008 (4)	Poultry workers, Guangdong China	Poultry-to-humans	2/231 subjects with "occupational exposure" had HI titers >1:80	Occupational exposure including raising, selling slaughtering chickens and ducks in H5N1 outbreak areas	Specific risk factors not identified
Cai et al., 2009 (5)	Firemen, government workers, vets for collection of dead wild birds on Ruegen Island, Germany 2006	Poultry-to-humans	0/97 workers were seropositive<br>Seropositivity by PN or MN assay if > 1:20	None	No evidence of H5N1 infection with subjects with exposure to infected wild birds; use of PPE was widespread
Wang et al 2009 (6)	Poultry workers in China 2007-2009	Poultry-to-humans	4/2191 using HI [no cutoff provided] had anti H5 antibodies	None	Limited evidence
Schultsz et al., 2009 (7)	Poultry workers and cullers living on farms with confirmed H5N1 outbreaks in poultry in Vietnam 04-05	Poultry-to-humans	0/500 (183 PW, 317 cullers) using MN and 3/500 (3 cullers) using HI >1:80 had anti H5 antibodies	Not evaluated	Limited evidence of poultry-to-human transmission despite exposure to infected poultry
Wang et al., 2009 (8)	Poultry workers in LBM in Guangzhou in 2006	Poultry-to-humans	0/68 were seropositive using HI [no cutoff provided]	None	No evidence of H5N1 infection with subjects with repeated exposure to infected poultry

Study, year	Study Population & Year of Outbreak	Transmission	Seroprevalence Results (% seropositive)	Risk Factors RR, OR, 95%CI	Comments
Occupationally Exposed Persons: Health Care Workers					
Bridges et al., 2000 (9)	Health care workers, Hong Kong 1997	Human-to-human; poultry-to-human	10/526 (8/21 exposed; 2/309 non exposed HCW) using MN >1:80, confirmed by WB	Bathing patients or changing the bed linen of cases (no OR provided); controlled for poultry exposure	Limited human-to-human transmission
Apisarnthanarak et al., 2005 (10)	Health care workers, Thailand 2004	Human-to-human; poultry-to-human	0/25 among HCW in direct contact with H5N1 patient; Seropositivity tested using MN >1:80, confirmed by WB	None	No serologic evidence of H5N1 among HCW with direct contact with human H5N1 patient
Thanh Liem et al., 2005 (11)	Health care workers, Vietnam 2004	Human-to-human; poultry-to-human	0/83 among HCW, 95% of which had direct contact with confirmed H5N1 patients Seropositivity tested using MN >1:40 in 2 independent assays	None	No serologic evidence of H5N1 among HCW with direct contact with human H5N1 patient
Schultsz et al., 2005 (12)	Health care workers, Vietnam 2004	Human-to-human; poultry-to-human	0/60 HCW in contact with confirmed H5N1 patients Seropositivity tested using MN >1:80 and ELISA >1:80	None	No serologic evidence of H5N1 among HCW with direct contact with human H5N1 patient
Non-Occupational Exposure: Household and Social Contacts					
Katz et al., 1999 (13)	Household and Social contacts of H5N1 patients, Hong Kong 1997	Human-to-human; poultry-to-human	6/51 (12%) household contacts 0/47 co-workers tested positive for H5 antibodies Seropositivity tested using MN or ELISA >1:80, confirmed by WB	None significant; however 21% of seropositive had contact to poultry vs. 5% of seropositive with no poultry contact, p=0.13	Human-to-human transmission was limited
Vong et al., 2006 (14)	Rural Cambodian villagers living in the same villages as two confirmed H5N1 human cases in 2005	Poultry-to-human	0/351 villagers tested positive for H5N1 antibodies ≥1:80 using MN and WB	None	No evidence of H5N1 infection among subjects living in villages with conformed H5N1 in domestic poultry flocks; poultry-to-human transmission was low in this setting
Lu et al., 2008 (4)	Poultry workers, Guangdong China	Poultry-to-humans	12/983 "general citizens" had HI or MN titers ≥1:20	Subjects were general citizens without direct contact with poultry	Specific risk factors not identified
Hinjoy et al. 2008 (15)	Rural poultry farmers in Thailand, 2004	Poultry-to-human	0/322 farmers tested positive for H5N1 antibodies; using MN >1:80, confirmed by WB or ELISA	None	No evidence of H5N1 infection among subjects living in villages with conformed H5N1 in domestic poultry flocks

Vong et al., 2009 (16)	Rural Cambodian villagers living in the same villages as confirmed H5N1 human case 2006	Poultry-to-human	7/674 (1%) seropositive for H5N1 antibodies using MN ≥1:80<br>85.7% (6/7) male<br>All ≤18 years old<br>Matched case-control study conducted with 7 seropositive cases and 24 controls	Swim/bathe in ponds OR 11.3 (1.25-102.2)<br>Water source 6.8 (0.68-66.4)<br>Gathered poultry and placed in cages or designated areas 5.8 (0.98-34.1)<br>Removed/cleaned feces from cages or poultry areas 5.0 (0.69-36.3)	Poultry-to-human transmission was low; possible transmission from the environment to humans via contaminated water
Dejpichal et al., 2009 (17)	Residents in 4 Thai villages with human cases in 2005	Poultry-to-human	0/901 tested positive for anti-H5 antibodies using MN confirmed by Immuno-<br>fluorescence >1:40	None	No evidence of H5N1 infection among subjects living in villages with conformed H5N1 in domestic poultry flocks
Santhia et al., 2009 (18)	Residents in 38 villages and 3 LBM in Bali, 2005	Poultry-to-human	0/841 tested positive for anti-H5N1 antibodies using MN >1:80	None	Despite H5N1 exposure from poultry outbreaks, no evidence of poultry-to-human transmission
Cavailler et al., 2010 (19)	Rural Cambodian villagers living in the same villages as confirmed H5N1 human case, 2007	Poultry-to-human	18/700 (2.8%) seropositive for H5N1 antibodies using MN ≥1:80	Swam/bathed in pond OR 2.52 (95%CI 0.98–6.51)<br>No other risk factors identified	Poultry-to-human transmission was low; possible transmission from the environment to humans via contaminated water

PPE = personal protective equipment including masks, gloves, eye protection; PW=Poultry workers; GW=government workers; HCW=health care workers
MN= microneutralization (MN) assay
HI = hemagglutination-inhibition assay
WB = Western Blot assay
PN = plaque neutralization

References

1. Bridges C, Lim W, Hu-Primmer J, Sims L, Fukuda K, Mak K, et al. Risk of influenza A (H5N1) infection among poultry workers, Hong Kong, 1997-1998. J Infect Dis2002;185:1005-10.

2. Wang M, Di B, Zhou D, Zheng B, Jing H, Lin Y, et al. Food Markets with Live Brids as Source of Avian Influenza. Emerg Infect Dis2006 November 2006;12(11).

3. Ortiz J, Katz M, Mahmoud M, Ahmed S, Bawa S, Farnon E, et al. Lack of Evidence of Animan-to-Human Transmission of Avian Influenza A (H5N1) Virus among Poultry Workers, Kano, Nigeria, 2006. 2007. p. 1685-91.

4. Lu C, Lu J, Chen W, Jiang L, Tan B, Ling W, et al. Potential infections of H5N1 and H9N2 avian influenza do exist in Guangdong populations of China. Chin Med J (Engl)2008;121(20):2050-3.

5. Cai W, Schweiger B, Buchholz U, Buda S, Littmann M, Heusler J, et al. Protective measures and H5N1-seroprevalence among personnel tasked with bird collection during an outbreak of avian influenza A/H5N1 in wild birds, Ruegen, Germany, 2006. BMC Infectious Diseases2009;9(1):170.

6. Wang M, Fu C, Zheng B. Antibodies against H5 and H9 avian influenza among poultry workers in China. N Engl J Med2009;360(24):2583-4.

7. Schultsz C, Van Dung N, Hai LT, Quang Ha D, Peiris JSM, Lim W, et al. Prevalence of Antibodies against Avian Influenza A (H5N1) Virus among Cullers and Poultry Workers in Ho Chi Minh City, 2005. PLoS One2009;4(11):e7948.

8. Wang Y, Liu Y, Jiang L, Liu Y, Yang Z, Hao A, et al. Risk assessment of H5N1 human infection after an outbreak of avian influenza in water fowl. Zhonghua Yu Fang Yi Xue Za Zhi2009 43(1):41-4.

9. Bridges C, Katz J, Seto W, Chan P, Tsang D, Ho W, et al. Risk of influenza A (H5N1) infection among health care workers exposed to patients with influenza A (H5N1), Hong Kong. J Infect Dis2000;181:344-8.

10. Apisarnthanarak A, Erb S, Stephenson I, Katz JM, Chittaganpitch M, Sangkitporn S, et al. Seroprevalence of Anti-H5 Antibody among Thai Health Care Workers after Exposure to Avian Influenza (H5N1) in a Tertiary Care Center. 2005. p. e16-e8.

11. Thanh Liem N, World Health Organization International Avian Influenza Investigation Team V, Lim W. Lack of H5N1 avian influenza transmission to hospital employees, Hanoi, 2004. Emerg Infect Dis2005;11(2):210-5.

12. Schultsz C, Vo C, Nguyen V, Nguyen T, Lim W, Tran T, et al. Avian Influenza H5N1 and Healthcare Workers. Emerg Infect Dis 2005;11(7):1158-9.

13. Katz JM, Wilina Lim, Bridges CB, Rowe T, Hu-Primmer J, Lu X, et al. Antibody Response in Individuals Infected with Avian Influenza A (H5N1) Viruses and Detection of Anti-H5 Antibody among Household and Social Contacts. The Journal of Infectious Diseases1999;180:1763–70.

14. Vong S, Goghlan B, Mardy S, Holl D, Seng H, Ly S, et al. Low Frequency of Avian-to-Human Transmission of H5N1 in Southern Cambodia, 2005. Emerg Infect Dis2006;Oct;12(10):1542-7.

15. Hinjoy S, Puthavathana P, Laosiritaworn Y, Limpakarnjanarat K, Pooruk P, Chuxnum P, et al. Low Frequency of Infection With Avian Influenza Virus (H5N1) Among Poultry Farmers, Thailand, 2004. Emerg Infect Dis2008;14(3):499-50.

16. Vong S, Ly S, Van Kerkhove MD, Achenbach J, Holl D, Buchy P, et al. Risk Factors Associated with Subclinical Human Infection with Avian Influenza A (H5N1) Virus- Cambodia, 2006. Journal of Infectious Diseases 2009;199(12):1744-52.

17. Dejpichai R, Laosiritaworn Y, Phuthavathana P, Uyeki T, O'Reilly M, Yampikulsakul N, et al. Seroprevalence of antibodies to avian influenza virus A (H5N1) among residents of villages with human cases, Thailand. Emerg Infect Dis2009;Available from http://www.cdc.gov/EID/content/15/5/756.htm.

18. Santhia K, Ramy A, Jayaningsih P, Samaan G, Putra A, Dibia N, et al. Avian influenza A H5N1 infections in Bali province, Indonesia: a behavioral, virological andseroepidemiological study. Influenza and Other Respiratory Diseases2009;3(3):81–9.

19. Cavailler P, Chu S, Ly S, Garcia J, Ha D, Bergeri I, et al. Seroprevalence of anti-H5 antibody in rural Cambodia, 2007. J Clin Virol 2010;Mar 29. [Epub ahead of print].
